# From Data to Decisions: Leveraging Artificial Intelligence and Machine Learning in Combating Antimicrobial Resistance – a Comprehensive Review

**DOI:** 10.1007/s10916-024-02089-5

**Published:** 2024-08-01

**Authors:** José M. Pérez de la Lastra, Samuel J. T. Wardell, Tarun Pal, Cesar de la Fuente-Nunez, Daniel Pletzer

**Affiliations:** 1 Biotechnology of Macromolecules, Instituto de Productos Naturales y Agrobiología, IPNA (CSIC), Avda. Astrofísico Francisco Sánchez, 3, 38206 San Cristóbal de la Laguna, (Santa Cruz de Tenerife), Spain; 2 Department of Microbiology and Immunology, School of Biomedical Sciences, University of Otago, 9054 Dunedin, New Zealand; 3 School of Bioengineering and Food Technology, Faculty of Applied Sciences and Biotechnology, Shoolini University, Solan, 173229 Himachal Pradesh India; 4 Machine Biology Group, Departments of Psychiatry and Microbiology, Institute for Biomedical Informatics, Institute for Translational Medicine and Therapeutics, Perelman School of Medicine, University of Pennsylvania, Philadelphia, PA USA; 5 Departments of Bioengineering and Chemical and Biomolecular Engineering, School of Engineering and Applied Science, University of Pennsylvania, Philadelphia, PA USA; 6 Department of Chemistry, School of Arts and Sciences, University of Pennsylvania, Philadelphia, PA USA; 7 Penn Institute for Computational Science, University of Pennsylvania, Philadelphia, PA USA

**Keywords:** AI/ML, Pathogen identification, Antibiotic resistance, Antibiotic stewardship, Personalized treatment, Antimicrobial peptides

## Abstract

The emergence of drug-resistant bacteria poses a significant challenge to modern medicine. In response, Artificial Intelligence (AI) and Machine Learning (ML) algorithms have emerged as powerful tools for combating antimicrobial resistance (AMR). This review aims to explore the role of AI/ML in AMR management, with a focus on identifying pathogens, understanding resistance patterns, predicting treatment outcomes, and discovering new antibiotic agents. Recent advancements in AI/ML have enabled the efficient analysis of large datasets, facilitating the reliable prediction of AMR trends and treatment responses with minimal human intervention. ML algorithms can analyze genomic data to identify genetic markers associated with antibiotic resistance, enabling the development of targeted treatment strategies. Additionally, AI/ML techniques show promise in optimizing drug administration and developing alternatives to traditional antibiotics. By analyzing patient data and clinical outcomes, these technologies can assist healthcare providers in diagnosing infections, evaluating their severity, and selecting appropriate antimicrobial therapies. While integration of AI/ML in clinical settings is still in its infancy, advancements in data quality and algorithm development suggest that widespread clinical adoption is forthcoming. In conclusion, AI/ML holds significant promise for improving AMR management and treatment outcome.

## Introduction

The discovery of penicillin marked a milestone in modern medicine, transforming the treatment of bacterial infections and establishing the golden era of antibiotic research [1]. However, widespread antibiotic use, both in human medicine and livestock production, has fueled the rise antibiotic resistance (AMR) [2, 3] globally. This poses a significant challenge in healthcare settings [4], particularly in Intensive Care Units (ICUs) [5], where multidrug resistant (MDR) pathogens are a major concern. Despite efforts in drug discovery, new antibiotic development has declined [6, 7], with current antibiotics insufficient to tackle AMR effectively [8], leading to high morbidity and mortality rates [4, 9].

Infectious disease management requires precise and rapid bacterial identification and antibiotic susceptibility testing for optimal patient outcomes [4, 5, 10, 11]. Recent advancements in artificial intelligence (AI) [12] have revolutionized various sectors, including healthcare, by analyzing extensive datasets, identify patterns and making predictions, eventually improving diagnostic accuracy [13, 14]. AI algorithms, particularly with machine learning (ML) capabilities, enables faster and more accurate diagnoses than conventional methods [15]. Integrating AI/ML into healthcare settings extends beyond predictive modeling to real-time monitoring, decision support systems, and drug discovery [16], facilitating proactive interventions and targeted antimicrobial stewardship [17, 18].

AI/ML is transforming drug discovery, particularly in antimicrobial peptides (AMPs) [12, 19], with potent antimicrobial properties. Computational modeling and predictive analytics accelerate AMP discovery and optimization, offering novel therapeutics against drug-resistant infections [20]. Continued advancements in AI/ML, combined with clinical expertise, hold promise in mitigating the impact of AMR and improving patient outcomes [17, 18].

### ML Methods in the Fight Against AMR

**Fig. 1:**
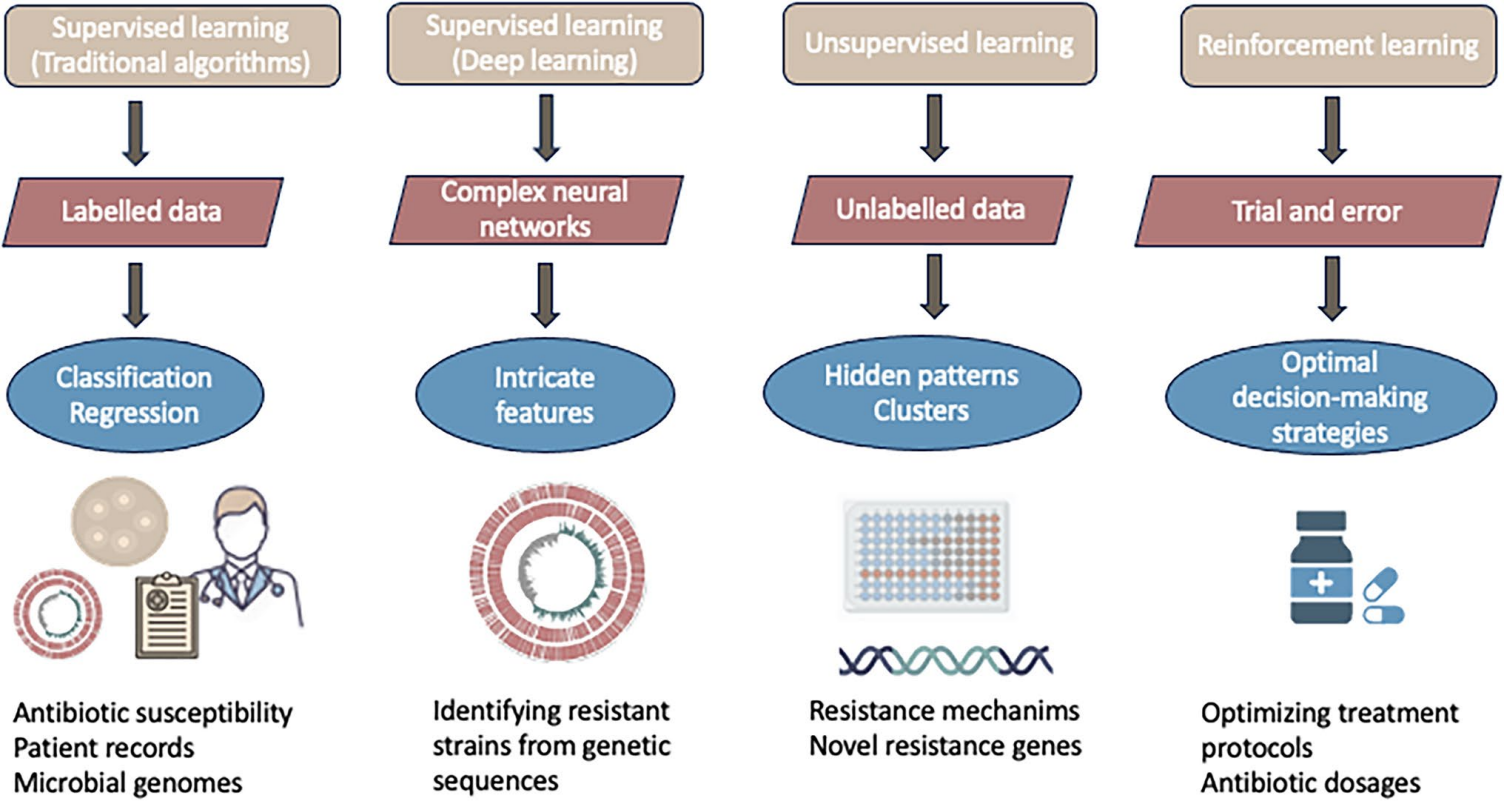
ML methods and applications in the fight against antimicrobial resistance

In the fight against antimicrobial resistance, ML offers a variety of techniques (Fig. 1) and applications (Table 1). Through computational modeling, virtual screening, and structure-based design, researchers can pinpoint potential drug targets, screen chemical compound libraries, and optimize lead candidates for antimicrobial activity. ML algorithms, trained on vast datasets of known antimicrobial agents, predict bioactivity, pharmacokinetic properties, and safety profiles of novel drug candidates, accelerating drug development and reducing time and costs associated with traditional approaches [21–23].

**Table 1:** Some examples of machine learning applications in the fight against antimicrobial resistance

**Pathogen/Topic**	**Application**	**Reference**
*Streptococcus pneumoniae*	Predict susceptibility to antibiotics	[24]
*Staphylococcus aureus*	Distinguish methicillin-resistant strains	[25]
*Escherichia coli*	Discover key genetic traits	[26]
	Optimize antibiotic treatment strategies	[34]
*Streptococcus pyogenes*	Prediction of virulence factors	[27]
*Salmonella enterica*	Investigate simultaneous presence of metal and antibiotic resistance	[30]
*Mycobacterium tuberculosis*	Predict resistance to specific antibiotics	[31]
Antibiotics	Predict antibiofilm activity	[28]
β-lactamases	Classify resistance or wild type	[29]
Drug discovery	Develop new antibacterial peptides	[32, 33]
Sepsis	Provide recommendations for antibiotic treatment	[35]

Supervised learning, a common ML method, predicts antibiotic sensitivity or treatment responses by training models on labeled datasets, such as microbial genomes or patient records (Fig. 1). As an example, it was used to predict the susceptibility of *Streptococcus pneumoniae* to β-lactam antibiotics by correlating penicillin-binding protein (PBP) sequences with minimal inhibitory concentration (MIC) values as labeled data. Additionally, sequences from the NCBI database that lacked MIC values were used as unlabeled data. This approach helped uncover the correlation between *S. pneumoniae* resistance phenotypes, serotypes, and sequence types [24]. Similarly, supervised ML identified genetic traits associated with antibiotic susceptibility in *Escherichia coli* across different sequence types (ST). These genetic markers help understand the development and spread of STs within clonal complexes that have a high transmission probabilities [26]. In a study on the prediction of virulence factors in *Streptococcus pyogenes*, López-Kleine et al. were able to narrow down the list of 1,507 genes to just 12 candidates without applying subjective filters or focusing on specific biological processes. These genes represent interesting targets for further biological validation and possible drug development [27]. Recently, a novel workflow utilizing ML to test genotype–phenotype associations has been proposed to improve the collection of high quality data on the virulence phenotype of *S. pyogenes* in conjunction with clinical outcomes [36].

Unsupervised learning analyzes unlabeled data to uncover hidden patterns or clusters among microbial populations, aiding the understanding of resistance mechanisms [37, 38] or discovery of new resistance genes (Fig. 1). Clustering algorithms, like K-means Clustering, group bacterial isolates based on resistance profiles or genetic characteristics [39]. A recent study classified β-lactamases as resistant or wild type, revealing distinct clusters with various strain characteristics [29]. Additionally, k-means clustering can assist in identifying novel resistance clusters or outbreaks, enabling timely interventions to mitigate the spread of antimicrobial-resistant infections. It has recently been used successfully to investigate the simultaneous presence of metal resistance and antibiotic resistance in *Salmonella enterica* [30].

Deep learning models, including convolutional neural networks (CNNs) and recurrent neural networks (RNNs) [40], extract complex features from genetic sequences to identify resistant strains or predict resistance mechanisms (Fig. 1). For example, CNNs accurately classify microbial strains as resistant or susceptible based on genomic sequences, while RNNs predict antimicrobial susceptibility from sequential data like treatment history or microbial evolution over time. Some CNN models have made it possible to identify functional genetic variations, provide physiologically relevant explanations, and have practical applications in clinical settings [40]. For example, a CNN was used to predict the resistance of *Mycobacterium tuberculosis* to 13 drugs by analyzing 18 specific sites in the genome previously not associated with resistance [31]. In addition, deep learning techniques such as generative adversarial networks (GANs) have shown promising results when used in the field of antimicrobial peptides [32]. GANs can develop new antibacterial peptides by changing the probability distribution of the generated sequences. Tucs et al. [33] generated six peptide variations with one of these peptides showing potent antibacterial activity against *Escherichia coli*.

Reinforcement learning (RL) trains algorithms to make decisions based on trial-and-error feedback, optimizing antibiotic treatment strategies or drug combinations against resistance [41] (Fig. 1). RL approaches optimize tasks with limited knowledge about system dynamics such as evolutionary simulations of bacterial populations. In a study using *E. coli* as a model, each genotype in the population was associated with a particular fitness landscape of the simulated evolution. The authors demonstrated that the reduction in population fitness due to the use of drug cycles was not constrained by an increase in genome size [34]. Recently, the RL approach has been shown to be useful in providing reasonable recommendations for antibiotic treatment in sepsis that are consistent with clinical practice [35].

Each ML methods offers unique advantages in tackling AMR, from identifying genetic markers and predicting resistance patterns to optimizing treatment strategies in real-time. The choice of methods depends on the specific problem and variables involved, ultimately improving efficiency and precision in developing new antimicrobial agents [42, 43].

### Antibiotic Stewardship

Antibiotic stewardship involves coordinated efforts to optimize antibiotic use, reduce unnecessary prescribing, and minimize the development of antibiotic resistance. It encompasses strategies at the institutional or healthcare system level to promote judicious antibiotic prescribing, optimize antibiotic selection and dosing, and prevent spread of multidrug-resistant organisms. While CDSS support individual clinical decisions, antibiotic stewardship programs address broader antibiotic use practices, aiming to maintain drug efficacy and combating antibiotic resistance [44]. AI systems enhance antibiotic stewardship in hospitals by monitoring antibiotic use and detecting overuse or inappropriate use. They analyze prescribing patterns, identifying anomalies [45], allowing targeted education or intervention to improve practices. They can be used to highlight situations where administered antibiotics do not match the approved first-line treatment or where broad-spectrum antibiotics are used without an explicit indication [46]. For example, a decision algorithm focused on urinary tract infections could reduce second-line antibiotics use by 67% compared to decisions made by clinicians, while decreasing inappropriate therapies by 18% [47].

AI analyzes microbiological data to detect evolving antibiotic resistance patterns, advising on antibiotic selection, dosage, duration, and de-escalation tactics based on individual patient data and antibiotic resistance trends. ML models, such as Extreme Gradient Boosting (XGB) and Light Gradient Boosting Machine (LGBM), discriminate scenarios requiring discontinuation of medication, transition of drug administration, and early or late reduction of antibiotic use, align well with clinical intuitions, resulting in improved efficiency [48]. This information helps to adapt antibiotic formularies or revise guidelines according to local resistance profiles. The integration of antibiotic stewardship into telemedicine with AI/ML technologies, transform outpatient healthcare. Telemedicine platforms, leveraging AI/ML algorithms, optimize antibiotic prescribing practices, improve clinical decision making, and ensure the judicious use of antibiotics [49]. A recent study compared telemedicine and in-person visits for acute respiratory tract infections found slightly higher guideline-concordant antibiotic management in telemedicine visits (92.5%) compared to in-person visits (90.7%). The findings suggest that with active antibiotic stewardship, telemedicine integrated into primary care can consistently deliver guideline-concordant care [50]. This integration addresses remote healthcare complexities and promotes effective antibiotic stewardship, leading to improved patient outcomes and sustainable antibiotic practices.

### ML for the Discovery of Novel Antibiotic Resistance Predictors

ML/AI approaches have emerged to address the ever-growing problem of antibiotic resistance. These technologies enable systems to analyze bacterial genomes, predict resistance, monitor epidemic patterns, and discover new antibacterial drugs or vaccines [51–57]. We refer to the extensive reviews by Anahtar et al. [55] or Wong et al. [23] that provide comprehensive overviews of ML application in the antimicrobial space.

Access to genome sequences and global surveillance data facilitates the prediction of antibiotic resistance based on genomic content, patient history, and infection characteristics [55]. ML excels at identifying factors contributing to resistance, such as resistance-associated genes [58], resistance-associated alleles [59], and treatment conditions [60], critical for optimizing therapies. Many studies now utilize ML algorithms to predict antibiotic resistance based on gene mutations, presence/absence of genes, and antibiotic sensitivities as data for training algorithms (Table 2). Many of these studies use rule-based or ML to predict the antibiotic resistance status of bacteria based on their treatment history, patient demographics, and genomic content. Promisingly, high prediction accuracies, often exceeding 90%, have been reported in various studies [61–63]. For example, Khaledi et al. [59] achieved high sensitivity (> 90%) in predicting resistance in *Pseudomonas aeruginosa* clinical isolates by using whole-genome sequencing (WGS) coupled with transcriptomics to identify a panel of biomarkers to make accurate predictions. Wang et al. [64] reported over 90% accuracy in predicting antibiotic resistance in *Staphylococcus aureus* bloodstream infections and obtained resistance predictions up to 6 h faster than traditional bacterial identification and antibiotic resistance testing methods.

**Table 2:** Overview of ML/AI methods using genomic information and antimicrobial sensitivity data for algorithm training

**ML/AI method(s)**	**Sample size**	**Sample type(s)**	**Year**	**Reference**
Logistic regression, decision trees, rule-based models	1393	Patient treatment history and patient demographics	2019	Cánovas-Segura et al. [65]
Decision support system	1886	Infection characteristics, patient treatment history, and patient demographics	2015	Chow et al. [66]
Support vector regression, cubist regression	382943	Patient antibiotic exposure history, infection characteristics, and patient demographics	2020	Chowdhury et al. [67]
Stack ensemble (through Azure AutoML)	11496	Antibiotic susceptibilities, patient demographics, and infection characteristics	2021	Feretzakis et al. [68]
Multiple methods tested using WEKA	23067	Antibiotic susceptibilities, patient demographics, and infection characteristics	2020	Feretzakis et al. [4]
Extreme gradient boosting	664	Antibiotic susceptibilities, whole genome sequencing, MALDI-TOF MS	2020	Ferreira et al. [69]
Support vector machine, random forests, logistic regression	414	Antibiotic susceptibilities, whole genome sequences, transcriptome sequencing, and geographic information	2020	Khaledi et al. [59]
Referenced-based Support vector machine, reference-free set covering machine	96	Antibiotic susceptibilities and whole genome sequences	2020	Liu et al. [53]
Logistic regression, K-nearest neighbor, random forest	144475	Patient demographics, infection characteristics, and antibiotic susceptibilities	2019	Martinez-Aguero et al. [52]
Reference-based kmer matching	1379	Antibiotic susceptibilities and whole genome sequences	2018	Mason et al*.* [70]
Extreme gradient boosting	68472	Antibiotic susceptibilities, patient demographics, and infection characteristics	2021	McGuire et al. [60]
Extreme gradient boosting	15580	Patient demographics and antibiotic susceptibilities	2020	Moran et al. [71]
Extreme gradient boosting	5278	Whole genome sequences and antibiotic susceptibilities	2019	Nguyen et al. [62]
Random forest	243	Patient demographics and antibiotic susceptibilities	2018	Oonsivilai et al. [51]
Gaussian naïve bayes, support vector machine, artificial neural network	15599	Patient demographics and infection characteristics	2021	Rawson et al. [72]
Logistic regression, support vector machine, random forest, convolutional neural network	9481	Antibiotic susceptibilities and whole genome sequences	2022	Ren et al. [58]
Logistic regression, Gaussian naïve bayes, support vector machine, decision trees, random forest, k-nearest neighbors, linear discriminant analysis, multinominal naïve bayes, AdaBoost classifier, gradient boosting classifier, ExtraTrees classifier, bagging classifier	3374	Antibiotic susceptibilities and Whole genome sequences	2021	Sunuwar et al.[73]
Logistic regression, decision trees	1618	Antibiotic susceptibilities, patient demographics, infection characteristics, and patient treatment histories	2017	Vazquez-Guillamet et al. [74]
Regression models	90000	Antibiotic susceptibilities, infection characteristics and patient treatment histories	2017	Wang et al. [75]
Random forest, support vector machine, k-nearest neighbors	125	Antibiotic susceptibilities, infection characteristics, and MALDI-TOF MS	2018	Wang et al. [76]
Convolutional neural network, random forest, extreme gradient boosting	12014	Antibiotic susceptibilities and MALDI-TOF MS	2022	Wang et al. [77]
Random forest, support vector machine	171	Antibiotic susceptibilities and MALDI-TOF MS	2022	Wang et al*.* [78]
Extreme gradient boosting, support vector machine, random forest	466	Antibiotic susceptibilities and whole genome sequencing	2022	Wang et al. [64]
Gradient boosting, logistic regression	711099	Antibiotic susceptibilities, patient demographics, infection characteristics, and treatment histories	2019	Yelin et al. [79]

Emerging technologies like matrix assisted laser desorption ionization-time of flight mass spectrometry (MALDI-TOF) mass spectrometry have further reduced the time to predict antibiotic resistance, leading to potential improvements in antibiotic stewardship and patient outcomes [80]. They also demonstrated in a retrospective analysis that by accelerating the prediction of antibiotic resistance using MALDI-TOF, 89% of their patients’ antibiotic regimens would have been changed, directly improving antibiotic stewardship, and potentially leading to measurable improvements in patient outcomes.

Moreover, ML is crucial in identifying epistatic interactions that can lead to resistance. For example, infectious diseases caused by *Mycobacteria*, *Pseudomonas*, and *Staphylococcus* all share a common problem: rifampicin resistance caused by missense mutations in the *rpoB* gene. These alterations reduce the nucleic acid affinity of the RNA polymerase complex by lowering the affinity of its constituent proteins. Portelli et al. have created a computational model that can predict whether or not a particular bacterial strain will develop resistance to the antibiotic rifampicin [81]. The discovery of these epistatic combinations or even rare mutations conferring resistance may be overlooked in current assessments, likely due to the need for prior knowledge of the genetics of resistance to be well understood for sequenced isolates to make accurate predictions. ML has been applied more frequently to predict antibiotic resistance caused by known resistance genes, or to identify genes whose role in resistance has been well characterized [59, 64, 82]. This requires a sufficiently large dataset of genomes and associated antibiotic susceptibility test results from both resistant and sensitive isolates to accurately train a de novo resistance prediction algorithm. The purpose of susceptibility testing is to determine whether antimicrobials suppress the growth of bacteria or fungi responsible for a particular disease. Combining WGS with routine antimicrobial susceptibility testing allows unprecedented mapping of genotype to phenotype. This information can then be used to train ML algorithms that can then be applied to new infections caused by the same organism to determine the most appropriate treatment. Clinically obtained isolates provide the most comprehensive examples to identify the spectrum of mutations that enable successful infection and have been used to inform ML algorithms [53, 58, 59, 62, 64, 73].

A potential disadvantage of using only clinical isolates to determine resistance is that a complex spectrum of mutations is found during infection [83–86] that may not necessarily be directly contributing to AMR. For example, mutations commonly identified in clinical isolates include genes involved in bacterial adaptation to the host’s hostile and changing environments, which can confound ML predictions. Frequently, mutations in clinical isolates are identified in genes that contribute to immune evasion, nutrient acquisition, metabolic shifts, reactive oxygen species (ROS) tolerance, production of extracellular polysaccharides to form biofilms, development of small colony variants, hypermutator strains, and ultimately AMR [83, 87, 88]. These mutations, which allow bacterial adaptation during infection, limit our ability to identify those genes directly contributing to AMR in vivo. The large genetic diversity of isolates can also lead to genetic noise. This genetic noise may attenuate the classification of predictors from the ML algorithms or identify predictors that are a byproduct of infection rather than resistance itself, as shown by studies that have found a number of predictors with no clear link to antibiotic resistance [89]. One possible means of combating genetic noise within clinical isolates is the use of in vitro evolution experiments against a variety of antibiotics. These experiments can be performed with a high number of replicates to identify the in vitro mutational spectrum on the selective pressure that contribute to AMR [90–94].

We believe that the genetic information identified by these experimental evolution studies could be used to train ML algorithms along with clinical isolates. Experimental evolution studies have already shown that many of the mutations identified in vitro are also selected for in naturally evolved clinical isolates [90, 95, 96]. ML has the unique ability to optimize its performance in real time by learning and incorporating new variables from microorganisms, patients, and antimicrobial agents.

### AI Systems to Assist in Selecting Appropriate Antimicrobial Therapy

AI systems can help clinicians select the most appropriate antimicrobial therapy by analyzing data and providing personalized treatment recommendations [17]. Clinical decision support systems (CDSS), integrated into electronic health records (EHRs), guide antibiotic selection, dosing, and duration based on patient-specific factors. CDSS have evolved considerably since their introduction in the 1980s, now providing real-time guidance through electronic medical records [97]. Retrospective analysis evaluates CDSS effectiveness, identifying areas for improvement and continuous enhancement [98, 99]. A recent study highlighted the utility of online CDSS in providing real-time surveillance data on antimicrobial resistance in community-acquired urinary tract infections (UTI). In this study, susceptibility profiles for *E. coli* were generated and compared with established surveillance systems. For complicated upper urinary tract infections, the CDSS point out the potential risks associated with the empirical use of fluoroquinolones and recommends considering the preferential use of third-generation cephalosporins based on current resistance patterns. The results showed significant differences in resistance rates between antibiotics, highlighting the potential of the CDSS to help clinicians select appropriate antimicrobial therapies based on current resistance patterns [100].

The RHINA system, a web-based CDSS, helps general practitioners in antibiotic prescriptions for rhinosinusitis. CDSS agreed with specialist decisions in over 90% of cases in a retrospective study with 1,465 patients, suggesting that such tools can reduce the over-prescription of antibiotics and consequently diminish bacterial resistance [101]. Recently, a retrospective cohort study examined the impact of pharmacist interventions triggered by a procalcitonin (PCT) on antibiotic use in patients with lower respiratory tract infections. Procalcitonin is a biomarker used to determine the presence of a bacterial infection and necessity of antibiotic therapy [102]. As a result of these pharmacist-led interventions, the total duration of antibiotic therapy in the hospital was reduced by 181 days. Of these, 125 days were directly attributable to reduced or optimized antibiotic therapy. In particular, vancomycin use decreased by 85.3% following the interventions compared to patients without documented interventions. The study suggests that pharmacist-led antimicrobial stewardship interventions guided by CDSS can effectively reduce antibiotic use and promote more targeted therapy, particularly in patients with normal PCT levels [103].

These examples illustrate the profound impact of AI and CDSS on enhancing antimicrobial therapy, optimizing treatment outcomes, and promoting better antibiotic stewardship practices.

### AI in Accelerating Antibiotic Discovery

**Fig. 2:**
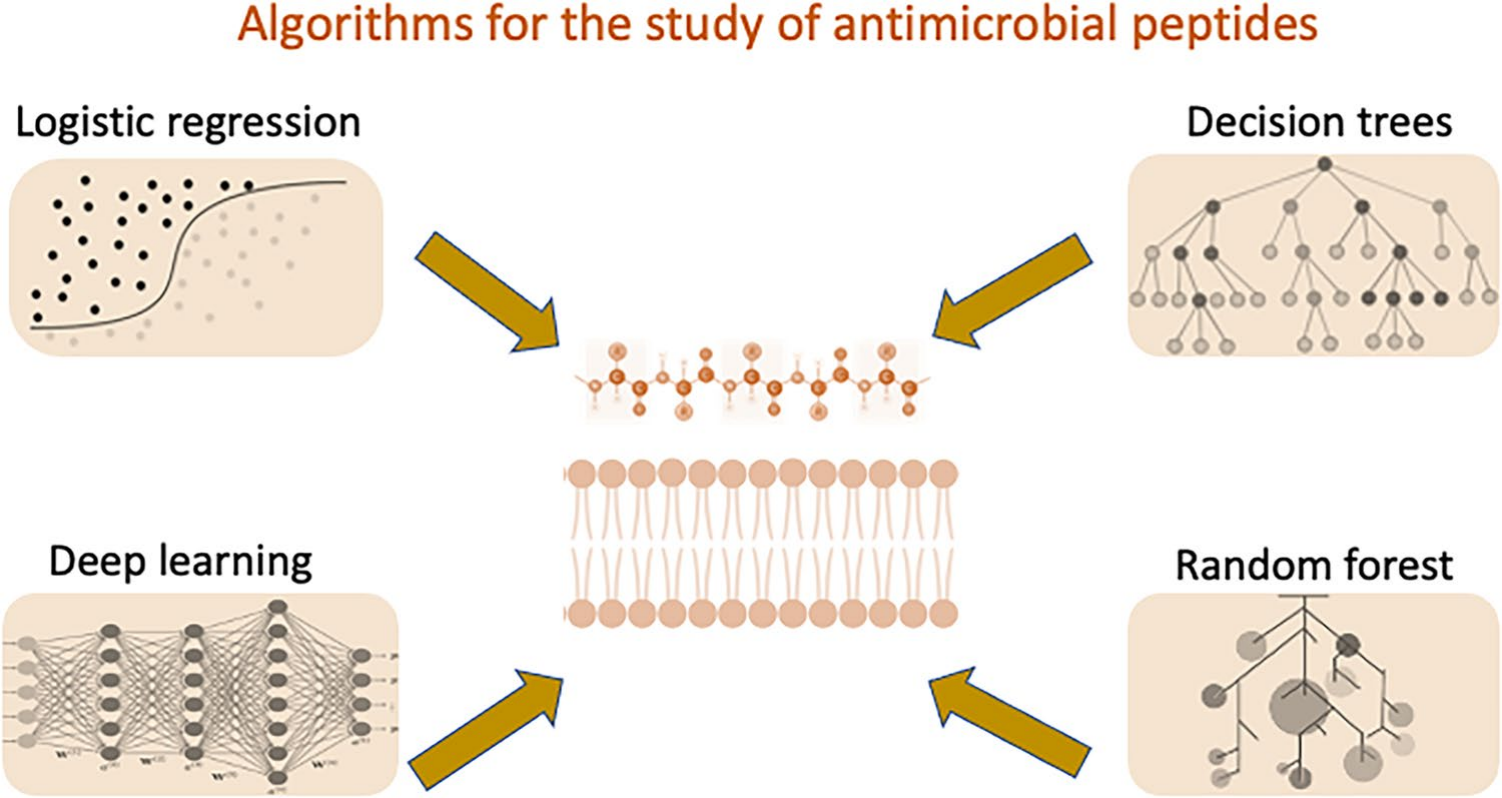
Common algorithms for antimicrobial peptide design and analysis

Even with efforts to identify antibiotic resistance patterns and ways to overcome or delay the ineffectiveness of drugs, it is only a matter of time before pathogens completely resistant to all antibiotics become ubiquitous. AI has become instrumental in accelerating the discovery of novel antimicrobial agents such as antimicrobial peptides (AMPs) [9, 12, 23, 104, 105]. AMPs are crucial components of innate host defense that show promise in penetrating bacterial membranes and inhibiting microbial growth. They offer a rich design space for targeted antimicrobial action [106]. Computational designs can predict novel AMPs from genome sequences, providing insight into chemical properties and bioactivities in AMP sequences (see detailed review by Wan et al. [12]). ML algorithms such as random forest, support vector machine (SVM) or logistic regression, decision trees, and deep learning [107] (Fig. 2), can predict antimicrobial properties from sequences [108]. For example, DL models trained with spider transcriptome data identified two peptides with potent antibacterial activity against various pathogens including *Bacillus subtilis, E. coli, P. aeruginosa, S. aureus, and S. epidermidis* [109]. Furthermore, new models using these algorithms aid in predicting various peptides activities beyond antimicrobial, potentially expediting drug discovery [110, 111].

ML can also be used to produce novel non-hemolytic AMPs. By training recurrent neural networks with data from the Database of Antimicrobial Activity and Structure of Peptides, Cappechi et al. [112] utilized recurrent neural networks to discover eight such AMPs effective against *P. aeruginosa, Acinetobacter baumannii*, and *methicillin-resistant S. aureus* (MRSA).

### Antimicrobial Peptide Development

We believe AMPs will be crucial in combating AMR. Key variables like net charge and pI are the most important variables underlying antibacterial activity of peptides. Söylemez et al. [113] established two accurate models for the classification of active antimicrobial peptides against (i) Gram-negative and (ii) Gram-positive bacteria. They found that net charge was the most important property for targeting Gram-negative bacteria, and pI for Gram-positive bacteria [113].

**Fig. 3:**
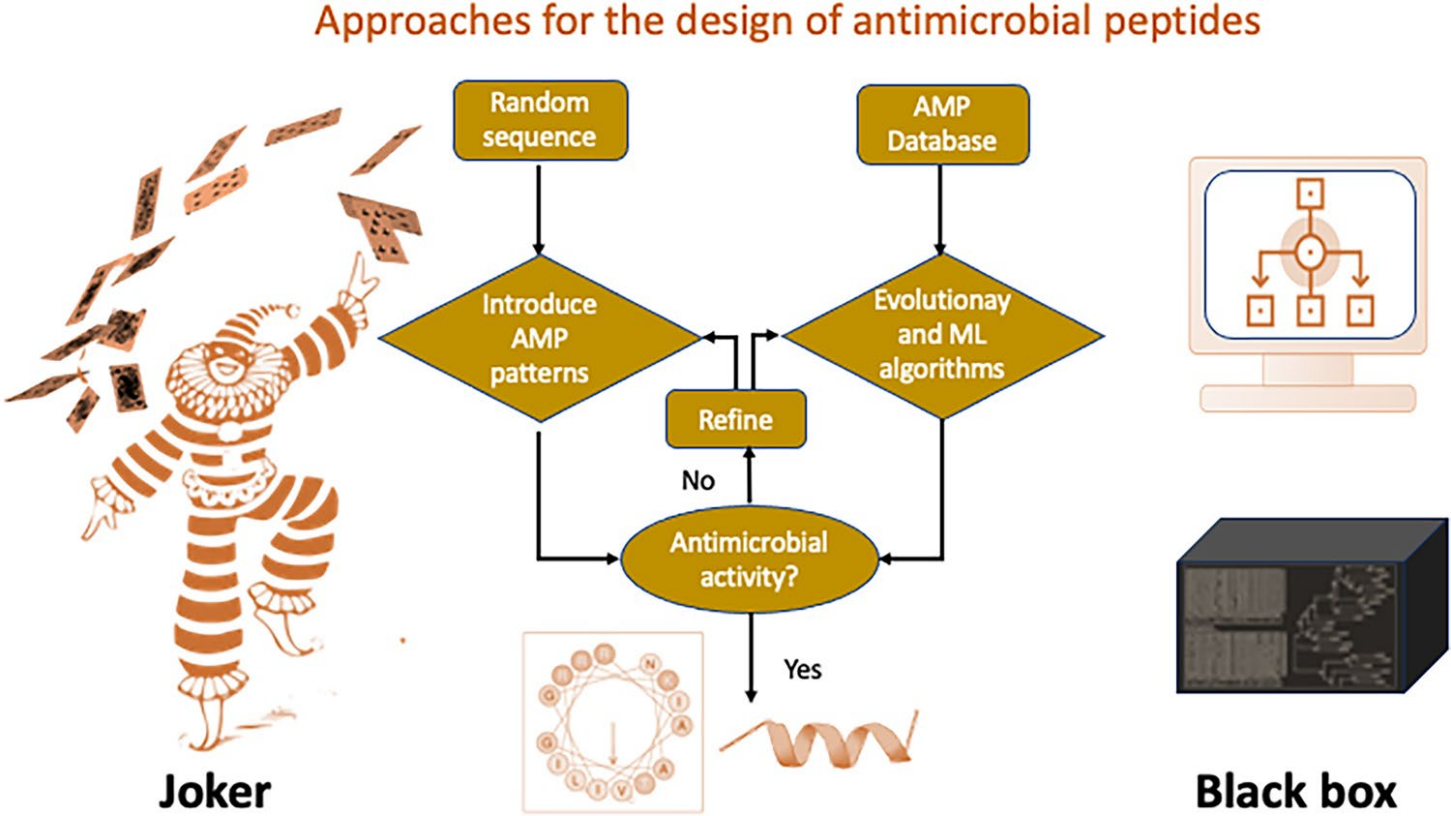
Key steps in the “joker” and “black box” approaches to antimicrobial peptide design

The “Joker” algorithm (Fig. 3) rapidly generates novel AMP sequences with broad-spectrum antimicrobial activity. Unlike targeting specific pathways or structures, “Joker” peptides utilize diverse physicochemical properties and structural motifs to disrupt bacterial membranes, inhibit essential cellular processes and evade resistance mechanisms. Due to their molecular diversity and promiscuity, "Joker" peptides exhibit robust activity against both Gram-positive and Gram-negative bacteria [114]. However, the versatile nature of "Joker" peptides poses a challenge in optimizing efficacy, selectivity and pharmacokinetic properties. Nevertheless, this approach led to the synthetic peptides PaDBS1R6 and EcDBS1R6, which reduced the viability of Gram-negative bacteria such as *P. aeruginosa* and *K. pneumoniae* [115, 116]. Another important strategy involves black-box ML methods that utilize sequence data patterns to describe antibacterial sequences (Fig. 3). For the black box algorithm, the flowchart might include selecting a database of antimicrobial peptides, training a machine learning model using the database, using the model to develop new peptides, testing the antimicrobial activity of the developed peptides, and refining the model and development process as needed. For the Joker algorithm, the flowchart could include selecting a random amino acid sequence, modifying peptide sequences using a sliding window, inserting an antimicrobial pattern into a set of target sequences by directly changing amino acids, testing the antimicrobial activity of the optimized sequence, and refining the sequence optimization process as needed.

In summary, the “Joker” and “Black Box” approaches offer complementary strategies in addressing antimicrobial resistance challenges. While the “Joker” emphasizes versatility and diversity, the “Black Box” prioritizes precision and rationality in optimizing antimicrobial activity and selectivity. By integrating both methodologies, researchers accelerate AMP discovery with improved efficacy, safety, and resistance profiles, advancing in antimicrobial therapy. Other major advances have also been recently described in this emerging field [117–120].

### AI and ML within the One Health Approach of Antibiotic Resistance

The One Health approach emphasizes the interconnectedness of human, animal, and environmental health, particularly regarding AMR. It underscores how antibiotic use in humans, animals and agriculture, contributes to the emergence and spread of resistant pathogens. The extensive use of antimicrobial drugs in livestock, both for prophylaxis and growth promotion, has raised concerns about the possible development of antibiotic resistance in humans [121], due to many organisms carried by livestock able to cause zoonotic diseases. Integrating AI/ML into the One Health efforts enables data-driven collaboration across various sectors to predict, monitor, and mitigate AMR risks effectively [122].

The concept of the exposome, introduced by Christopher Wild in 2005, is crucial for understanding AMR transmission. It encompasses all environmental exposures throughout a person’s life, including antimicrobial use in healthcare, agriculture, and the environment. AI/ML techniques analyze exposome-related datasets to identify patterns linking environmental factors and AMR. By integrating clinical records, environmental monitoring, and genomic sequencing data, these models pinpoint risk factors and predict future AMR trends. Combining big data analysis, ML algorithms, and Geographical Information Systems (GIS) enhances one-health risks assessment and environmental management within the One Health framework [123].

### Advantages and Limitations of AI/ML in the Fight Against AMR

AI and ML offer numerous advantages in the fight against AMR by integrating various types of data (genomic, phenotypic, clinical and epidemiologic) to enhance predictive models [124]. These technologies enable early detection of rising resistance, allowing for proactive intervention. AI/ML streamline the search for resistance mechanisms and therapies, reducing the need for manual testing and minimizing human error. Advanced algorithms and simulations help identify new pharmacological and therapeutic targets, improving our ability to combat AMR [125, 126]. In addition, AI/ML facilitate the customization of antibiotic treatments based on individual patient and pathogen data. These technologies promote multidisciplinary collaboration among computer scientists, biologists, physicians and other experts, fostering comprehensive and innovative solutions to antibiotic resistance challenges [127] (Table 3). While AI/ML shows promise in tackling AMR, several limitations hinder its broad application in this field (Table 3). Access to comprehensive datasets containing diverse genomic, phenotypic, clinical, and epidemiological information remains a challenge, impacting the accuracy of AI predictions [128, 129]. Data quality issues, such as incomplete or biased data, can further compromise algorithm performance [130]. Additionally, AI models, particularly deep learning ones, often lack transparency, operate as “black boxes”, hindering interpretability in clinical settings [131]. Regulatory and ethical considerations, including patient safety and data privacy, also pose challenges to AI integration [132]. Moreover, the dynamic nature of microbial evolution and resistance mechanisms, presents difficulties for AI adaptation and generalization across diverse settings and pathogens [133]. Despite these hurdles, interdisciplinary collaboration and ongoing research hold promise for overcoming these barriers and leveraging AI to advance prescind medicine, optimize antibiotic use, and combat AMR effectively.

**Table 3:** Advantages and limitations of AI/ML approaches for antimicrobial resistance

**Advantages**	**Limitations**
Encourages multidisciplinary collaboration between computer scientists, biologists, physicians, and others to address AMR problems	Requires extensive, high-quality, and comprehensive data sets, which can be difficult to obtain and integrate
Combines diverse data sources (genomic, phenotypic, clinical, and population) to enhance prediction models	Ethical and regulatory barriers, such as privacy and patient safety concerns, may hinder the adoption and use of these technologies
Facilitates early detection of increasing resistance, allowing for proactive intervention	The dynamic nature of microbial evolution necessitates constant updating and retraining of models, which can quickly become outdated
Accelerates the search for resistance mechanisms and therapies, reducing the need for manual testing and minimizing human error	Complex models and large data sets require significant computing resources and expertise from technology specialists
Identifies new pharmacological and therapeutic targets to combat AMR using advanced algorithms and simulations	Training data may introduce biases that influence the model, and the models may not fully capture complex biological relationships
Customizes antibiotic treatment based on individual patient and pathogen data	High development and implementation costs of AI-based diagnostic systems may limit accessibility in low- and middle-income countries

## Data Availability

No datasets were generated or analysed during the current study.
